# Children’s Paradise

**Published:** 1899-08

**Authors:** M. F. Underwood

**Affiliations:** San Francisco


### CHILDREN'S PARADISE.

M. F. Underwood, M. D., San Francisco.

A city of one-third of a million inhabitants with only 240 deaths from children's diseases is the astonishing figure given in the report of the Board of Health of San Francisco for the past year, classified as follows: Cholera infantum, 47; infantile diarrhœa, 7; measles, 1; diphtheria, 154; pertussis, 25; thrush, 1; croup, 5.

From the above it will be seen that the death rate from intestinal diseases was almost nil. The statement that only forty-seven children died in this city of cholera infantum during the whole year is almost beyond credit to one who has practiced in other climates; but such is the phenomenal truth. In fact, cholera infantum is so seldom met with that it is commonly said that children are exempt from it in California. Only five deaths from croup, and that terribly fatal disease, membranous croup, had not a single victim. What a contrast to the record of cities in other parts of the world!

It is on this account that so many people of means bring their children here to give them, during the first years of their life, this almost entire immunity from those diseases which bring down such a large percentage of children as their prey.

Scarcely a single ward in New York, Chicago, St. Louis, or New Orleans can show so few deaths from these diseases as this whole city; and it pleases me to say that not a single death occurred here when pure Homœopathy was employed.

Children's diseases of all kinds are very light here; even diphtheria is much less severe than in other localities, making California as it is truly named, "The Children's Paradise." The bracing pure air, blowing from the northwest, laden with ozone and cool ocean vapor, furnishes such a vigorous atmosphere that sudden changes seldom occur and the vitality is kept at such a normal gauge that little can come to in any way overthrow the equilibrium of the life-force of the child.

The soil, too, being so sandy and of such great depth, and so dry and porous, is also a source of both health and happiness to the little ones. The governess, however, has many sad experiences in trying to keep her charge from its normal mischief, namely, "rolling in the nice, clean dirt."

Children love the earth, and to deprive them of either joy or benefit of "playing in the dirt" is scarcely pardonable.

I have often wished for a sort of children's home where pale, anæmic children of the city could be taken and turned loose in brown duck, with little spades and hoes and buckets and encouraged to indulge to their hearts' content. It would be better than medicine to the little pale residents of the crowded streets of our cities. Why don't these philanthropic millionaires institute such things for the poor children instead of stylish asylums by which to advertise their generosity?

In many a back yard in this city will be found a box about a foot deep and say ten feet square, filled with clean, white ocean sand. This is for the children to play in, and great frolic they have, too, with this wise provision. Ocean sand being as clean as grass, and impregnated as it is with the elements of the sea, is more than mere sand.

While I am writing this (July 7th) the temperature is sixty-two degrees F. and the same weight of clothing is being worn as in the holidays, and with the same comfort. The cool, bracing summers, and almost the same thermometer the year round gives to the young children such a start in the race against disease that they grow up into the most perfect types of manhood and womanhood to be found in this country.

